# Implementing public health and social measures: an integral part of the health emergency management cycle

**DOI:** 10.1093/eurpub/ckac129.535

**Published:** 2022-10-25

**Authors:** J Addo, D Cocciolone, C Gapp, A Latta, S Lindmark, L Owen, J Sane, I Perehinets, T Schmidt, C Wippel

**Affiliations:** Country Health Emergency Preparedness and IHR, WHO Regional Office for Europe, Copenhagen, Denmark

## Abstract

Public health and social measures (PHSM) have been utilized as a tool to reduce the infection rates and disease burden throughout the COVID-19 pandemic and continue to play an important role even with vaccination campaigns well underway in preventing severe disease. In order to systematically track, analyze and report qualitative and quantitative data o PHSM implementation across the European Region and assist countries in the COVID-19 response, the COVID-19 Incident Management Support team at the WHO Regional Office for Europe developed PHSM Severity Index and PHSM Dashboard. The PHSM Severity Index captures the types, severity and timing of PHSMs implemented by a country across six main indicators. By providing standardized data on PHSM implementation, the PHSM Severity Index can support and inform the development of policy at country and regional levels. PHSM data, severity methodology and policy tools developed throughout the COVID-19 pandemic should be adapted to provide a foundation for preparedness for future large-scale health emergencies. In addition, discerning the epidemiological impact of specific PHSM and their combinations currently is a priority for policy-makers and can guide countries’ transition strategies. Analysing the impact of PHSM on COVID-19 transmission is of critical importance, especially as variants of concern bring new waves of COVID-19 cases and may challenge countries’ vaccination and response strategies. Reference PHSM in Response to COVID-19 (who.int)

